# MiR-21 regulating PVT1/PTEN/IL-17 axis towards the treatment of infectious diabetic wound healing by modified GO-derived biomaterial in mouse models

**DOI:** 10.1186/s12951-022-01516-4

**Published:** 2022-06-28

**Authors:** Xi Chen, Yizhong Peng, Hang Xue, Guohui Liu, Ning Wang, Zengwu Shao

**Affiliations:** 1 Department of Orthopeadics, Union Hospital, Tongji Medical College, Huazhong University of Science and Technology, Wuhan, 430022 Hubei China; 2 National Engineering Research Center of Fiber Optic Sensing Technology and Networks, Wuhan University of Technology, Wuhan, 430070 China

**Keywords:** Diabetic wound, miR-21-5p, PVT1, Graphene oxide, PDLLA-PEG-PDLLA

## Abstract

**Background:**

Diabetic foot ulcer (DFU), persistent hyperglycemia and inflammation, together with impaired nutrient and oxygen deficiency, can present abnormal angiogenesis following tissue injury such that these tissues fail to heal properly. It is critical to design a new treatment method for DFU patients with a distinct biomechanism that is more effective than current treatment regimens.

**Method:**

Graphene oxide (GO) was combined with a biocompatible polymer as a kind of modified GO-based hydrogel. The characterization of our biomaterial was measured in vitro. The repair efficiency of the biomaterial was evaluated in the mouse full-skin defect models. The key axis related to diabetic wound (DW) was identified and investigated using bioinformatics analyses and practical experiments.

**Result:**

In the study, we found that our modified GO-based wound dressing material is a promising option for diabetic wound. Secondly, our biomaterial could enhance the secretion of small EVs (sEVs) with more miR-21 by adipose-derived mesenchymal stem cells (AD-MSCs). Thirdly, the PVT1/PTEN/IL-17 axis was found to be decreased to promote DFU wound healing by modifying miR-21 with the discovery of PVT1 as a critical LncRNA by bioinformatics analysis and tests.

**Conclusion:**

These findings could aid in the development of clinical care strategies for DFU wounds.

**Supplementary Information:**

The online version contains supplementary material available at 10.1186/s12951-022-01516-4.

## Introduction

The formation of a diabetic foot ulcer (DFU) is a leading cause of morbidity and death in patients with poorly managed diabetes [1–4], with an estimated 25% of diabetes patients suffering from chronic infectious wounds that fail to properly heal and are associated with a risk of amputation, and the 5-year survival rate with such non-resolving wounds is estimated to be only 32% [5]. By preventing infection, eliminating injured tissue, and secreting cytokines that stimulate fibroblast migration, angiogenesis, collagen deposition, and wound closure, wound healing could be restored and facilitated [6, 7]. Among those above-mentioned risk factors, the systemic abnormalities in normal angiogenesis and vascular growth could hamper diabetic wound healing, resulting from damaged tissues that are insufficiently oxygenated and deprived of essential nutrients [8, 9]. So far, there has not been any satisfactory solution.

Adipose-derived mesenchymal stem cells (AD-MSCs) are a more effective source of stem cells with a higher capacity for migration, proliferation, and differentiation, compared with bone marrow-derived MSCs (BM-MSCs) [10–12]. Benefiting from their paracrine secretion, AD-MSCs are enabled to initiate a repairing process of progenitor-cell populations, such as upregulating immune response and proangiogenic factors [13, 14]. Extracellular vesicles (EVs), derived from MSCs and used in tissue engineering, particularly for reducing inflammation and promoting angiogenesis and vascular development, may represent a new direction in recent years [15, 16]. EVs could transfer non-coding microRNAs (miRNAs) to recipient cells to aid wound healing by enhancing the communication of cell-to-cell and cell-to-other miRNAs, proteins, and growth factors [17, 18]. It is reported that skin wound healing is delayed in miR-223^Y/−^ mice with prolonged neutrophil activation and IL-6 expression [19]. Small EVs (sEVs) secreted by AD-MSCs could promote angiogenesis by suppressing DLL4 by transferring miR-125a [20]. However, since the whole picture of the precise molecular mechanism has not been clearly revealed yet and side effects or other adverse reactions are not under control, it is extremely urgent for people to exploit a new option with a clear biomechanism [21].

Biomaterials have been extensively studied recently, they could be empowered with different functions, such as catalytic hydrogen evolution reaction (HER) performance, photothermal activity, superior conductivity, special superaerophobicity and superhydrophilicity [22–28]. A number of different novel biomaterials could be potential candidates to date for use in wound dressings designed to treat cases of chronic diabetic wound [29–31]. Zhao et al. found that a hydrogel called PDA@Ag NPs/CPHs had a noticeable effect on DFU by increasing angiogenesis, speeding collagen deposition, and inhibiting bacterial growth [32]. Graphene oxidation yields graphene oxide (GO) nanosheets, which, owing to their high biocompatibility and bioavailability, have been developed to generate promising compounds or nanoparticle-mediated drug delivery applications [33, 34]. Most of them contain metal icons like the chemical compound ZIF-8@GOx@BHb within Zn, which could enhance peroxidase activity by producing gluconic acid [35]. Although their biocompatibility was well exhibited, how they facilitate cell proliferation and inhibit cell apoptosis is not fully explicit. Furthermore, because there are disadvantages to clinical wound dressing, it is essential to develop a new form to fulfill market expectations.

In this study, we found that our modified GO-based wound dressing material is a promising candidate for diabetic wounds (DW). Secondly, our biomaterial could enhance the secretion of sEVs with more miR-21 by AD-MSCs. Thirdly, the PVT1/PTEN/IL-17 axis was found to be decreased to promote DFU wound healing by modifying miR-21 with the discovery of PVT1 as a critical LncRNA by bioinformatics analysis and tests.

## Results

### PEP@GO characterization

**Fig. 1:**
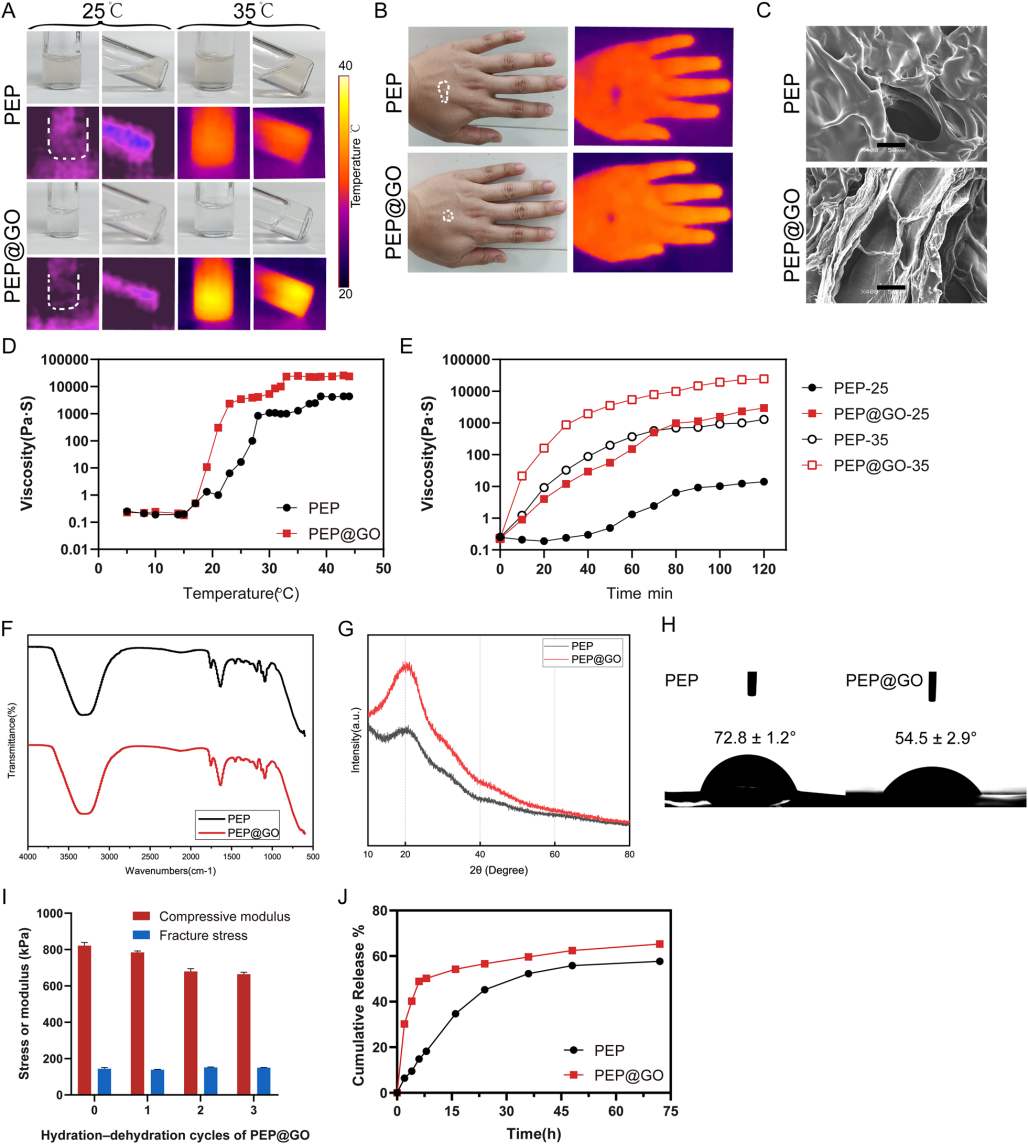
Characterization tests for PEP and PEP@GO. **A** Digital and infrared thermal images of liquid-gel phase transition during heating and cooling (25–35 ℃). **B** Digital and infrared thermal pictures on the handback of human skin at RT. **C** High-resolution images, scale bar: 50 μm and **D** viscosity curves at different temperatures and **E** over time. **F** FTIR spectra. **G** XRD patterns. **H** Water contact angle. **I** Compressive modulus and fracture stress of PEP@GO after different hydration-dehydration cycles. **J** Cumulative release curves at 37 ℃

The PEP and PEP@GO rheological characteristics were first evaluated to determine their mechanisms and reversibility. These hydrogels exhibited a liquid to sol–gel phase transition during cooling and heating (25–35 ℃) as observed with an infrared thermal imaging system, with both of these solutions remaining in a liquid state at 25 ℃ but undergoing gelation following immersion in a 35 ℃-water bath. The PEP@GO exhibited greater solidity than did the PEP hydrogel, especially when the bottle was oblique, suggesting that the PEP@GO exhibits superior adhesion and is better suited to practical clinical utilization (Fig. 1A).

To further assess hydrogel adhesion stability on human skin in situ, 200 µL of hydrogel were placed on the human hand’s back and turned vertical for 10 min at RT to observe vertical shifts under the influence of gravity. The PEP@GO attached to the skin under these conditions, maintaining a stable shape whereas the other fell down. Interestingly, the temperature of PEP@GO was closer to skin temperature than PEP after 10 min, consistent with better thermal conductivity (Fig. 1B). The PEP@GO created on silicon plate could practically maintain structure at an ambient temperature of around 30 °C, as shown in Additional file 1: Fig. S1A, whereas the PEP hydrogel transitioned to liquid without the assistance of a heat source.

The SEM images indicated that PEP harbored a regular, porous structure, with PEP@GO containing even more pores as well as small rough granular inclusions (p = 0.0013). GO was observed on the surface of these PEP@GO, consistent with effective GO adherence thereto (Fig. 1C). Although there was little difference between the two hydrogels, the viscosity of PEP@GO increased much faster than PEP from 25 to 35 ℃, and they both tended to be stable after reaching 35 ℃. The PEP@GO showed more viscous at last, the viscosity data remained nearly constant after attaining the maximum value at roughly 32 °C (Fig. 1D), and the rheological properties of both hydrogels increased over time (Fig. 1E). FTIR spectra analyses were conducted to assess the surface chemical properties of PEP and PEP@GO. In the resultant analysis (Fig. 1F), the peaks respectively at 3312.16 and 3328.22 cm^−1^ (–CH_3_), 1753.30 and 1754.71 cm^−1^ (C=O), and 1090.26 and 1092.07 cm^−1^ (C–O) were consistent with the expected composition of these biomaterials [36]. Additional file 1: Fig. S1B showed that the uniformity of PEP@GO was satisfied. No apparent changes in PEP@GO consistent with a chemical reaction and appearance were observed relative to PEP, suggesting that GO was merely dispersed within these PEP preparations via cross-linking without any likely impact on the functional properties of these materials. The X-ray diffraction (XRD) patterns of both hydrogels showed that the peak was around 2θ = 20°, and after that they both decreased to the same level as the degree increases (Fig. 1G). The broad peak represented a typical diffraction pattern of hydrogel. The broad spectrum around 2θ = 20° displayed by the diffraction spectrum of PEP showed its amorphous nature. To examine the change in wetting of the micro carrier’s surface, we evaluated the water contact angle of two hydrogel surfaces. The contact angle on PEP was 72.8 ± 1.2°, and on PEP@GO was 54.5 ± 2.9°, the latter was smaller with better wettability (Fig. 1H). When it comes to interacting with cells, the hydrophilicity of the materials is critical. The mechanical characteristics of PEP@GO are shown in Fig. 1I. After three hydration-dehydration cycles, the compressive fracture stress and compressive modulus of the hydrogel were only slightly changed after three hydration–dehydration cycles. The release curves showed that, as the temperature increased, the PEP@GO was released very quickly at first, reaching 60% after 72 h, while the other one reached 50% (Fig. 1J).

### In vitro and in vivo analyses of PEP@GO antibacterial activity and biocompatibility

**Fig. 2:**
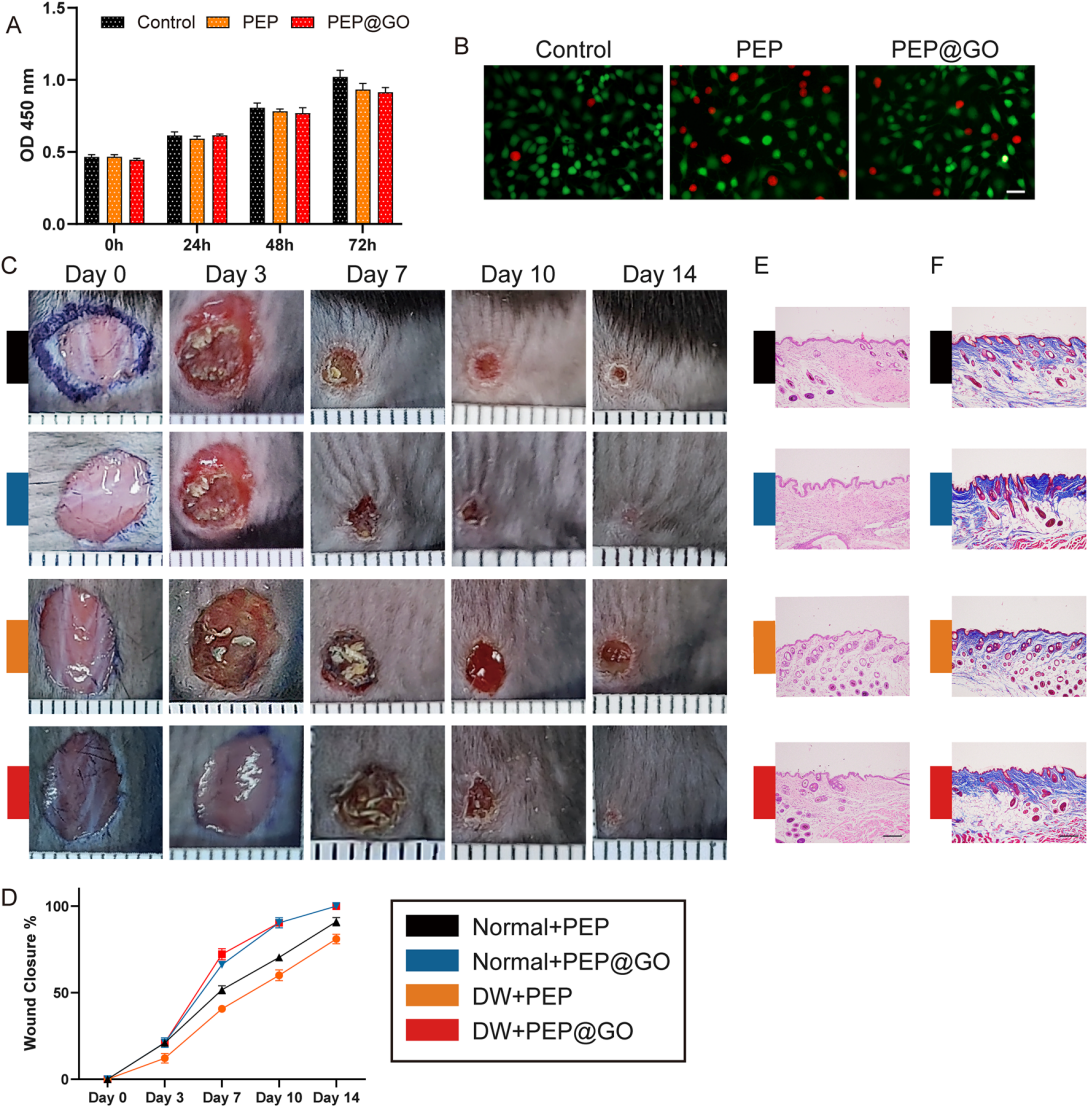
Biocompatibility of PEP@GO compared with PEP and control group. **A** CCK-8 assay results of HUVEC proliferation after treatments at 0, 24, 48 and 72 h. **B** Live/Dead staining of HUVECs cultured on day 1. Green and red fluorescent cells are viable and dead, respectively. Scale bar: 50 μm. **C**, **D** Normal and HG were constructed on the back skin of each mouse. Digital pictures were collected at Day 0, 3, 7, 10 and 14, and its statistical data, minor ticks’ interval: 1 mm. **E** H&E staining and **F** Masson staining images of skin section collected from wound area of each group at Day 14, scale bar: 200 μm. Data are the means ± SD three independent experiments. *p < 0.05, **p < 0.01, ***p < 0.001

Next, CCK-8 assays and LIVE/DEAD staining assays were then conducted to assess the biocompatibility (Fig. 2A, B), revealing that PEP@GO treatment had no adverse impact on HUVEC growth. These results suggested that our prepared hydrogel represented a promising agent that was unlikely to inadvertently harm healthy cells.

To expand upon the above findings, we used a cutaneous wound healing model to assess the in vivo functionality of prepared PEP@GO. This analysis revealed that PEP@GO application markedly accelerated DW healing and epidermal regeneration relative to other tested treatments, with an increase in the wound closure ratio and better healing after a 14-day period (Fig. 2C, D). Subsequent H&E (Fig. 2E) and Masson’s staining (Fig. 2F) of these wound tissues revealed the presence of an intact epithelial barrier and extensive collagen production in samples from mice in the two groups involved with PEP@GO, with minimal inflammatory cell infiltration, whereas samples from the other two groups exhibited extensive inflammatory cell infiltration, few collagen fibers, and a disordered epithelial barrier. Together, these data showed that in vivo our PEP@GO exhibited superior activity in promoting DW healing and could more efficiently repair damaged skin barriers.

### Bioinformatics analyses of high throughput sequencing data

**Fig. 3:**
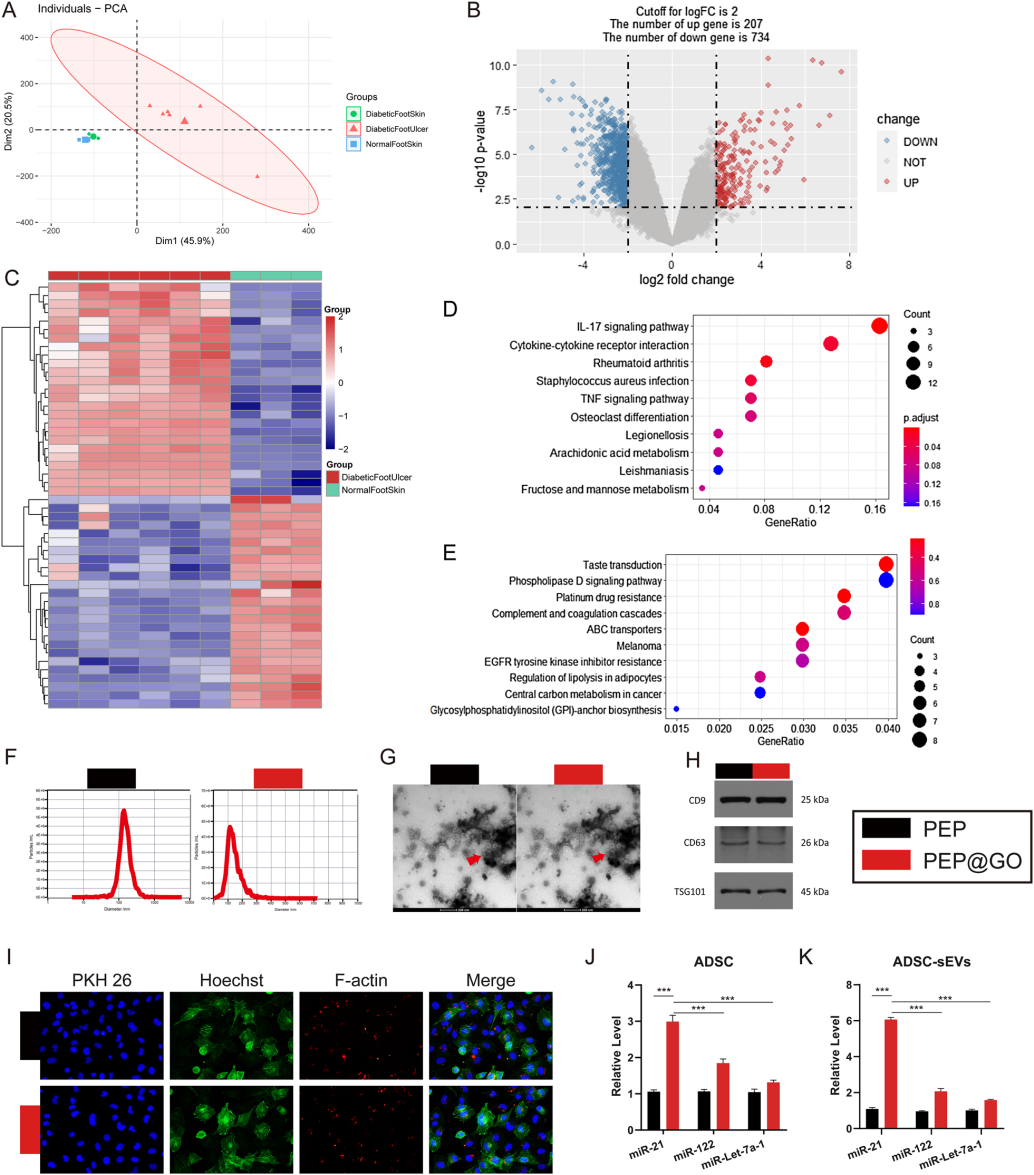
Bioinformatics analysis of expression profiling by high throughput sequencing on GSE80178 and regulation of miR-21 by PEP@GO **A** PCA of hierarchical cluster analysis of all the 12 samples. **B** Volcano plot for DEGs between the DFU and normal samples. Red and blue dots represent up- and down-regulated genes, respectively, while grey are no significantly differentially expressed genes. **C** Heatmap of top 25 up- (red) and down-regulated (blue) genes in the DFU and Normal samples, and the color depth of the blocks represents the degree of mediation. **D**, **E** KEGG pathway enrichment analysis of infection-associated (**D**) up- and (**E**) down-regulated pathways. The color of the dots increases from blue to red to indicate the significance of enrichment gradually, and the number of differential genes found in each pathway is indicated by the size of the dots. Characterization of sEVs stimulated by PEP and PEP@GO (**F**–**I**). **F**, **G** The mean particle size distribution of PEP-sEVs and PEP@GO-sEVs. **G** TEM pictures of the ultrastructure of the two kinds of sEVs. Scale bar: 200 nm. **H** The marker protein levels of CD9, CD63, and TSG 101. **I** The sEVs of two groups marked with the red fluorescence dye PKH26 were co-cultured with HUVECs Scale bar: 50 μm. qPCR results of relative level of three miRNAs in ADSCs after co-cultured with PEP@GO and PEP (**J**) and in sEVs of two groups (**K**)

First, Principal Component Analysis (PCA) and the hierarchically clustered heatmap analyses of the top 100 upregulated genes in the 12 analyzed samples revealed a clear differentiation between DFU samples and samples in other groups, suggesting that skin damage and ulceration were associated with differences in gene expression (Fig. 3A). The DEGs between DFU and normal control tissue samples were then assessed, with a total of 911 DEGs being identified from 53,617 total genes, of which 207 were upregulated and 734 were downregulated at the selected cutoff criteria (logFC = 2) (Fig. 3B). Heatmaps of the top 25 up- and down-regulated genes were additionally generated (Fig. 3C).

To determine the specific changed genes in the whole course of disease, the functional relevance of identified DEGs was assessed through GO and KEGG analyses performed with the R “clusterProfiler” package. As KEGG pathway analyses showed, upregulated genes were found to be enriched in infection-related pathways such as the IL-17 signaling pathway, S. aureus infection, and TNF signaling pathway (Fig. 3D), whereas downregulated genes were found to be enriched in the Phospholipase D signaling and EGFR tyrosine kinase inhibitor resistance pathways (Fig. 3E), indicating an intense inflammatory response and inhibition of wound recovery.

The lists’ intersections from GeneCards led to the identification of 6 miRNAs of potential relevance (Additional file 1: Table S1), with miR-21 exhibiting the highest GeneCards Inferred Functionality Scores (GIFtS) and Relevance Scores of 25/3.92 and 24/16.18 for the two keywords. Similar polymer biomaterials containing GO have been reported to promote miR-21-5p expression [37]. We assumed this miRNA may have a potential role in promoting healing activity of the prepared PEP@GO. Enrichment of miR-21-5p in DFU was down-regulated compared with that in normal (Additional file 1: Fig. S2A). The core miR-21-5p and wound target genes were obtained for GSEA analyses and used to generate protein–protein interaction networks with the STRING tool (string-db.org, v11.0) and Cytoscape 3.82 (Combined Scores ≥ 0.8). PIK3R-1 was the only gene associated with both of these pathways, and was thus a central hub gene (Additional file 1: Fig. S2B, C). The PI3K/Akt signaling pathway can promote tissue repair and reverse IL-17-induced pathological inflammation, in which PIK3R-1 is vital in this pathway [38].

These bioinformatic analyses thus revealed that our graphene oxide-derived biomaterial was able to promote miR-21-5p upregulation via upregulating the PI3K/Akt signaling pathway during the DW healing. Next, we sought to confirm these results through a series of experiments.

### ADSC-sEVs preparation and analysis

After isolation, sEVs were assessed via TEM, DLS, and Western blotting. DLS analyses indicated that these particles ranged from 50 to 150 nm in size (Fig. 3F), while TEM confirmed the presence of round, lipid bilayer-enclosed particles 50–200 nm in diameter (Fig. 3G), in line with prior reports [39]. The sEVs’ surface markers such as CD9, CD63, and TSG101 were found on these particles (Fig. 3H). HUVEC uptake tests were used in additional in vitro and in vivo experiments. HUVECs were able to pick up both normal and diabetic sEVs in 12 h, as evidenced by the red fluorescence staining in the cells (Fig. 3I).

To determine whether PEP@GO can promote high-content miR-21 vesicles, ADSCs were cultured with PEP@GO and PEP, and miR-21 was quantified using qPCR. As shown in Fig. 3J, K, the PEP@GO relative level of the selected top three miRNAs was increased in both ADSCs after co-culture with sEVs in the supernatant, with miR-21 growing the fastest (p < 0.0001), followed by miR-122 and miR-Let-7a-1. When compared to ADSCs (Fig. 3K), miR-21 in PEP@GO sEVs (Fig. 3J) increased significantly more than in the PEP group.

### Assessment of the effects of miR-21-5p on the treatment of HUVECs

**Fig. 4:**
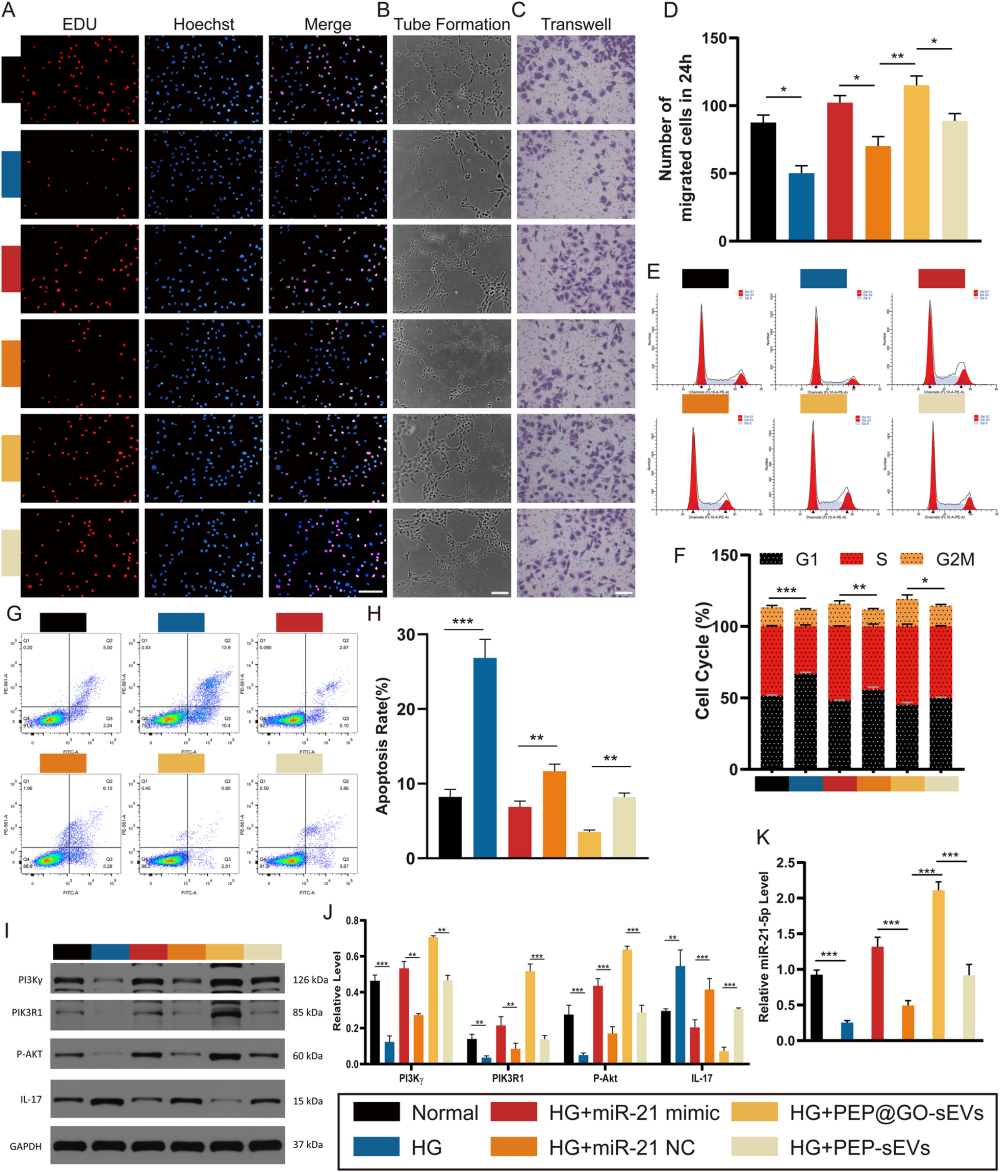
The regulation of PEP@GO-sEVs and miR-21 mimic in the treatment of HUVECs with HG*.* HUVECs were randomly distributed into six groups with different treatments. **A** EDU incorporation assays, **B** tube formation assays, **C** transwell migrate on assays and **D** its statistical data, **E**–**H** cell cycle distribution and rates of apoptosis in different groups by flow cytometry, **I** Western Blotting results of key proteins such as PI3Kγ, PIK3R1, P-Akt, and IL-17 in six groups and **J** its statistical data, **K** qPCR results of relative miR-21-5p level in the tissues of the six groups, scale bar: 100 μm. *p < 0.05, **p < 0.01, ***p < 0.001

The effects of ADSC-sEVs triggered by PEP@GO on the DFU healing process were then investigated. Therefore, HUVEC proliferation, migration, and tube formation were therefore analyzed, revealing that the proliferation of HUVECs in normal control, high glucose (HG) + miR-21 mimic, and HG + PEP@GO-sEVs was significantly increased relative to the other three groups, respectively. PEP@GO-sEVs were able to protect against the negative effects of HG treatment (Fig. 4A), with similar outcomes being observed in tube formation assays (Fig. 4B) and transwell migration assays (Fig. 4C, D). Cell cycle distributions and apoptosis rates in different groups were quantified by flow cytometry, revealing a significantly increased proportion of S phase cells in the HG + PEP@GO-sEVs group compared with the HG + PEP-sEVs group, with a corresponding decrease in the apoptosis rate. When HUVECs were treated with both HG and PEP@GO, the rate of apoptosis was reduced relative to the HG group. Interestingly, there was a markedly increased apoptosis rate in the HG group (Fig. 4E–H). These findings suggested that HG could promote apoptosis and disrupt proliferation, migration, and angiogenesis, while PEP@GO-sEVs could protect against these changes.

Subsequent western blotting assays indicated that HUVECs in the normal, HG + miR-21 mimic, and HG + PEP@GO-sEVs groups exhibited significant increases in PI3K/Akt pathway protein levels, including PI3Kγ, PIK3R1, and p-Akt relative to cells in the HG, HG + miR-21 NC and HG + PEP-sEVs groups. In addition, levels of PI3Kγ, PIK3R1, and p-Akt in the HG group was reduced relative to the HG + PEP-sEVs and HG + PEP@GO-sEVs groups. The opposite changes were observed in the IL-17 expression (Fig. 4I, J).

Next, qPCR assays were conducted to assess miR-21-5p levels in these treatment groups (Fig. 4K), revealing it to be expressed at the highest levels in the HG + PEP@GO-sEVs group whereas the HG group exhibited the lowest levels. Therefore, ADSC-sEVs addition in the presence of PEP@GO was associated with further increases in miR-21-5p expression.

The above in vitro results indicate that, with the treatment of PEP@GO, the PI3K/Akt signaling pathway was up-regulated while the IL-17 pathway was down-regulated*.* And similar results could be seen with miR-21-5p.

### Diabetic wound healing assay regulated by PEP@GO and miR-21

**Fig. 5:**
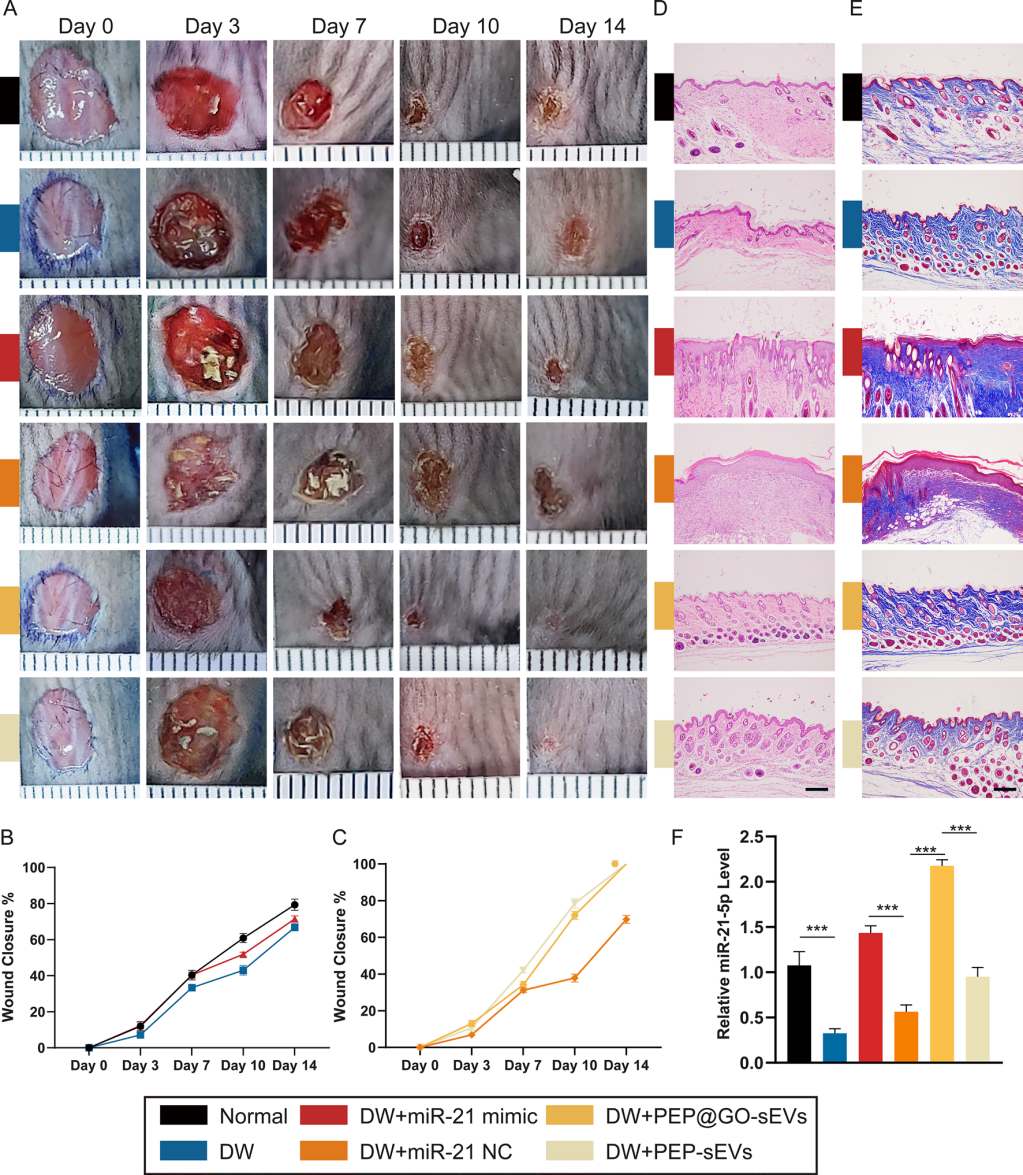
Wound healing assays were constructed on the back skin of each mouse in the six groups. **A**–**C** Representative digital pictures were collected at Day 0, 3, 7, 10 and 14 in the wound healing process and its statistical data, minor ticks’ interval: 1 mm. **D** H&E staining. **E** Masson staining images of skin sections of the six groups at Day 14. Scale bar: 200 μm. **F** qPCR results of the relative miR-21-5p level in the tissues of the six groups at Day 14. *p < 0.05, **p < 0.01, ***p < 0.001

Assays assessing the effects of miR-21-5p and hydrogel-associated sEVs on cutaneous wound healing were next conducted (Fig. 5A–C), revealing that PEP@GO application markedly enhanced such epidermal regeneration (PEP@GO-sEVs vs PEP-sEVs), whereas the application of DW in isolation delayed wound healing, as shown by comparing the HG vs the normal, and the opposite situation could be seen in DW + miR-21 mimic vs DW + miR-21 NC. In histological images of H&E (Fig. 5D) and Masson’s (Fig. 5E) stained wound tissues collected on day 14, an intact epithelial barrier and collagen fibers were evident in the PEP@GO-sEVs group with minimal inflammatory cell infiltration, whereas extensive inflammation, barrier disruption, and few collagen fibers were evident in the DW + miR-21 NC and HG groups. However, the application of PEP@GO-sEVs was sufficient to reverse the DW on membrane integrity, underscoring the value of this hydrogel in the context of wound healing. When back tissue samples isolated from these animals on day 14 were assessed via qPCR, we found that miR-21-5p was upregulated in the PEP@GO-sEVs group compared with the PEP-sEVs group (Fig. 5F) in accordance with before (Fig. 4K). Then we looked at the particular ways that miR-21-5p was linked to wound healing.

### MiR-21-5p could bind to PTEN and PVT1

**Fig. 6:**
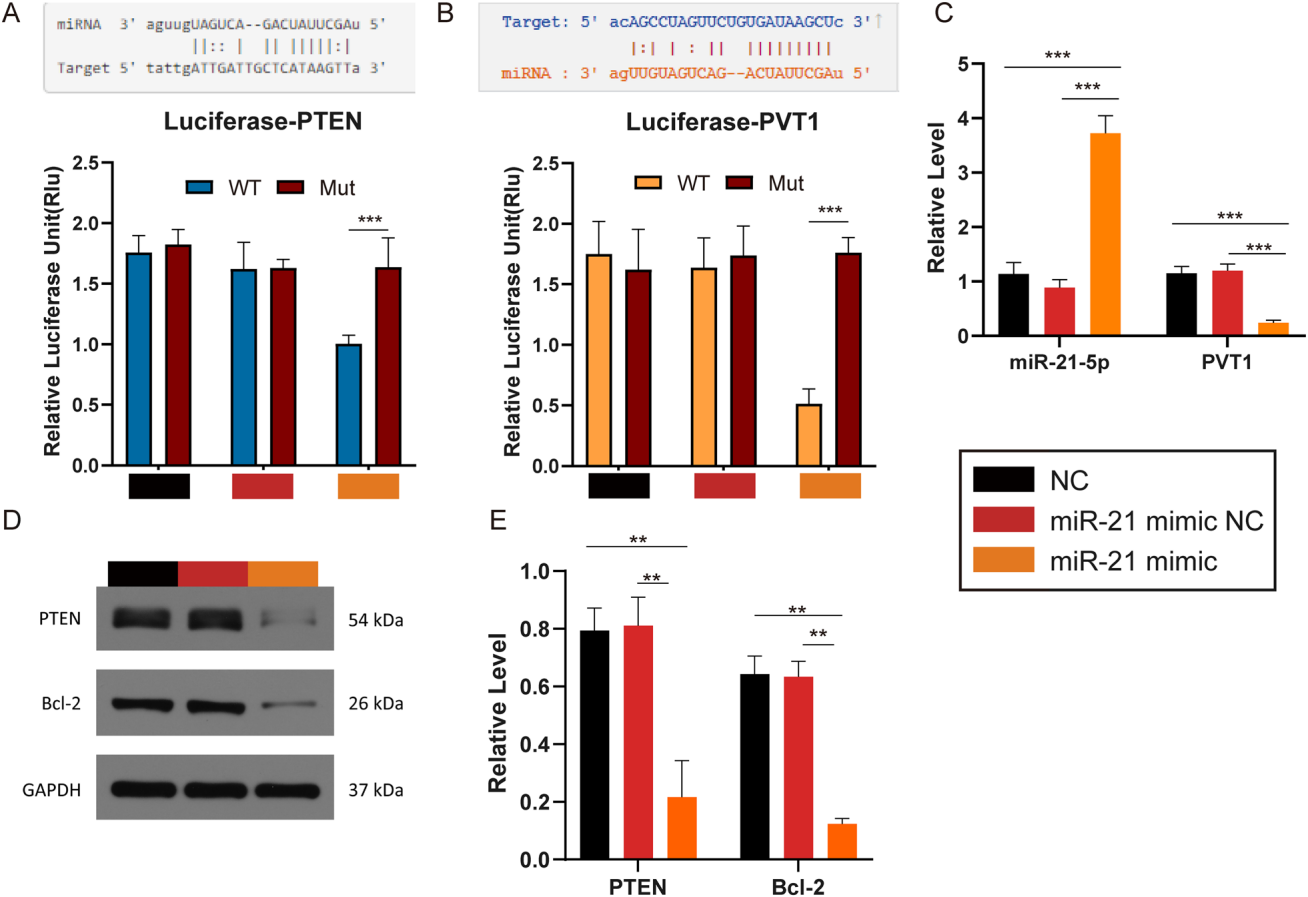
Regulation of miR-21-5p on PTEN and PVT1*.* HUVECs were randomly distributed into three groups that received different treatments. **A**, **B** Predicted binding sites between miR-21-5p and PTEN and PVT1 respectively by the database (starbase.sysu.edu.cn/; mirtarbase.mbc.nctu.edu.tw/) and relative Luciferase unit of three groups treated with the wild type (WT) or Mutated (Mut) versions of **A** PTEN and **B** PVT1. **C** qPCR results of relative miR-21-5p and PVT1 levels. **D** Western Blotting results of key proteins such as PI3Kγ, PIK3R1, P-Akt, IL-17 and **E** its statistical data. *p < 0.05, **p < 0.01, ***p < 0.001

In subsequent luciferase reporter assays, miR-21-5p mimic transfection suppressed PTEN WT and PVT1 WT reporter activity (Fig. 6A, B). These findings revealed that miR-21-5p suppressed the expression of PTEN and PVT1 by binding to them. The miR-21-5p levels were found to be higher in the miR-21-5p mimic groups with no significant differences in the NC groups, whereas PVT1 levels were lower (Fig. 6C).

We next transfected HUVECs with miR-21 mimic constructs to see if they could target genes related to PTEN. The western blotting revealed that the negative control constructs had no impact on protein levels in treated cells, whereas miR-21 overexpression was associated with reductions in PTEN and Bcl-2 expression (Fig. 6D, E).

### PVT1 competitively binds miR-21-5p and thereby impacts fibroblast proliferation, migration, and tube formation

**Fig. 7:**
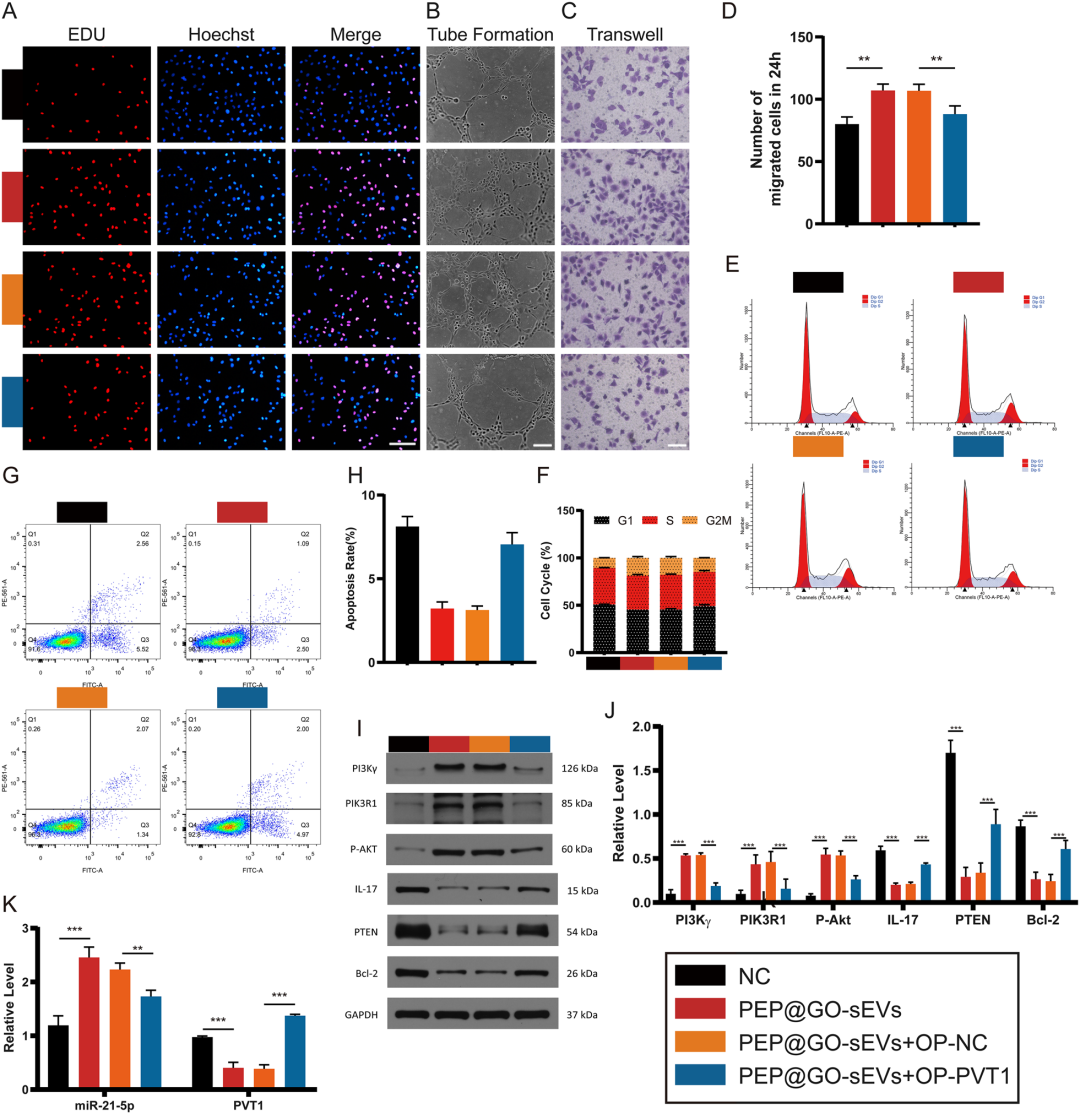
The impact of PEP@GO-sEVs and PVT1 on HUVEC functionality*.* HUVECs were randomly distributed into four groups with different treatments. **A** EDU incorporation assays, **B** tube formation assays, **C** transwell migration assays and **D** its statistical data, **E**–**H** cell cycle distribution and rates of apoptosis by flow cytometry, **I** Western Blotting results of key proteins such as PI3Kγ, PIK3R1, P-Akt, IL-17 and **J** its statistical data, **K** qPCR results of relative miR-21-5p and PVT1 level, scale bar: 100 μm. *p < 0.05, **p < 0.01, ***p < 0.001

To verify the ability of PVT1 to competitively bind to miR-21-5p, plasmids were generated and transfected into HUVECs. The pcDNA3.1 vector was imported into the RNA mature sequence of PVT1 for lncRNA (OP-PVT1). Following that, EDU assays revealed that PEP@GO-sEVs + OP-PVT1 transfection decreased HUVEC proliferation. However, cells treated with PEP@GO-sEVs constructs showed no such effect (Fig. 7A). Similarly, the involvement of PEP@GO-sEVs + OP-PVT1 was also observed in both tube formation (Fig. 7B) and transwell migration assays (Fig. 7C, D). Next, flow cytometry was performed, revealing a significantly decreased proportion of S phase cells after treatment with PVT1 relative to the other three groups together with an increased apoptosis rate (Fig. 7E–H). These findings suggested that PVT1 can suppress proliferation, migration, and angiogenesis while facilitating apoptosis.

Next, the western blotting assays revealed significant decreases in the expression of PI3Kγ, PIK3R1, and p-Akt in the PI3K/Akt pathway following PEP@GO-sEVs + OP-PVT1 transfection. While PEP@GO-sEVs were added, these levels were increased whereas IL-17, PTEN, and Bcl-2 levels were decreased (Fig. 7I, J). Next, qPCR analyses were used to assess relationships between miR-21-5p and PVT1 (Fig. 7K), indicating that PVT1 suppressed miR-21-5p expression.

The findings showed that miR-21-5p might interact directly with PTEN and PVT1, with PVT1 competitively binding miR-21-5p, preventing it from influencing PI3K/Akt signaling pathway activity and thereby inhibiting HUVEC proliferation.

### Assessment of PEP@GO biotoxicity

Histological examinations of important organs such as the heart, liver, spleen, lung, and kidney were used to determine the long-term biocompatibility of PEP@GO (Additional file 1: Fig. S3A). No apparent lesions or pathological findings were evident in any of these tissues following H&E staining. Routine hematological analyses of these mice were also conducted, revealing no significant changes in any analyzed parameters or white blood cell (WBC) counts, consistent with a lack of any PEP@GO-induced damage (Additional file 1: Fig. S3B). There were also no changes in hemoglobin (HGB) or platelet (PLT) levels, consistent with any hydrogel-related hemolysis or coagulopathy (Additional file 1: Fig. S3C, D).

## Discussion

**Fig. 8:**
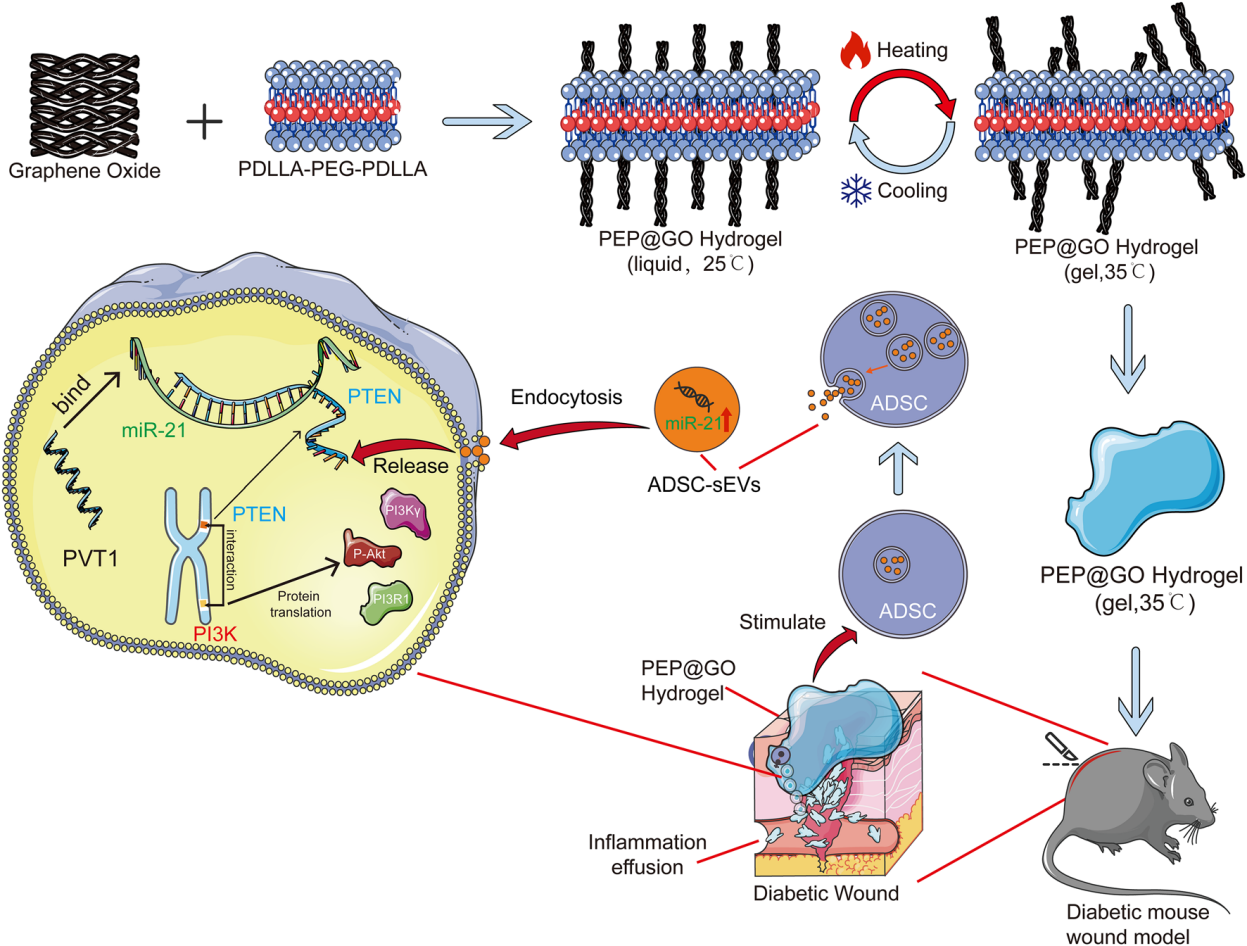
Schematic structures of temperature-sensitive PEP@GO, and its abstract that promotes diabetic wound healing via up-regulating miR-21 in ADSC

Herein, we successfully prepared and characterized a novel temperature-sensitive, porous PEP@GO capable of promoting wound healing in a DFU model system. Our biomaterial could enhance the secretion of sEVs with more miR-21 by AD-MSCs. When the biomaterials were involved, the PVT1/PTEN/IL-17 axis was found to be decreased to promote DFU wound healing by modifying miR-21 with the discovery of PVT1 as a critical LncRNA by bioinformatics analysis and tests, making it a promising tool for DFU treatment (Fig. 8).

A number of surfaces have been conjugated or otherwise coated with GO, including cotton and polymer film, thereby protecting the underlying substrate while promoting wound healing efficacy [40]. A sprayable hydrogel composed of a copolymer and Ag@rGO nanosheets was shown to be temperature-responsive and to effectively form a stable in situ wound dressing [41]. Unlike the above material, without any metal involved, our PEP scaffold used for our biocompatible hydrogel nanomaterial exhibits advantageous biological properties while effectively synergizing with GO to yield a temperature-responsive biomaterial. Owing to its well-defined structure, PEG allows for effective reaction tuning and for the stoichiometric incorporation of bio-functional groups for in situ gelation in vivo [42]. The features not only keep the material attached to the wound in solid form but also take advantage of the preservation.

As targets of active biological research, miRNAs are essential non-coding RNAs that play essential roles in regulating processes including development, differentiation, immunity, and reproduction [43]. MiR-21 could be delivered to macrophages using targeted nanoparticles in the context of impaired diabetic wound healing by repairing those tissue disruptions through the silencing of both KLF-5 and PTEN [44]. Modulating miR-21 expression could improve wound healing and reduce inflammatory responses [45]. MiR-21 also plays an important role in human mesenchymal stem cell-derived sEVs effects on target cells, increasing calcium handling via PI3K signaling [46]. Interestingly, in our results, the group with PEP@GO-sEVs showed the best performance compared with others, including those with miR-21 mimic. The sEVs’ plasma membrane-like phospholipids and membrane-anchored proteins, unlike plasmids, liposomes, and other carriers, may contribute to their reduced clearance from the circulation [47, 48]. As another key part, the LncRNA PVT1 could sustain tissue homeostasis by being adjacent to the oncogene MYC in the skin epidermis [49]. With the assistance of bioinformatic data and practical experiments, we identified that miR-21 could bind to PVT1 at a specific site, eliminating the influence of damaged skin without self-repairing capability, and our biomaterial could trigger the antimicrobial pathway via miR-21 and PVT1.

To further illustrate the mechanisms that were involved in our materials in the regulation of diabetic wound healing, the PI3K/Akt signaling pathway could reverse the effects of IL-17 at the level of GSK-3b, while also ensuring appropriate checkpoint vessel formation and improving damaged microcirculation in the context of DFU [38, 50]. The phospholipase D1 pathway, as a downstream target of the Wnt/β-catenin and PI3K/Akt pathways, can influence the expression of proliferation-related targets such as Raf-1 and ERK and survival-related targets such as mTOR [51, 52]. These signaling pathways could play a critical role in the mechanisms whereby our PEP@GO facilitates DFU healing. Moreover, PEP@GO could stimulate ADSC to generate sEVs with more miR-21 with the help of GO by comparison with PEP, followed by greater activation of downstream pathways, including the PI3K/Akt signaling pathway, consistent with our high throughput analysis and experimental findings.

This study has several limitations. Firstly, large DFU sample analysis, which this work did not have, could be more faithful and accurate. Secondly, while the database and transcriptome sequencing used in our study could provide a good aim to explore the mechanism, but more precise will be applied in future research. Thirdly, our exploration of a key pathway involved in miR-21 in the wound healing process could be more evidential if we verified it in vivo.

## Conclusion

In summary, we have advanced current knowledge regarding the mechanisms of DFU by treating GO-derived biomaterials, indicating that biomaterials could be an option for the clinical management of DFU. As further research continues, it will be of great significance for other various non-healing wounds with different dangers that might be effectively managed. These findings could aid in the development of clinical care strategies for DFU wounds.

## Materials and methods

### Preparation of GO

GO was prepared using the modified Hummer’s method [17]. In brief, graphite (10 g, 80 mesh, 94% carbon) was combined with NaNO_3_ (9 g) and concentrated H_2_SO_4_ (350 mL) at room temperature (RT) for 3 h before being chilled in an ice bath and treated with KMnO_4_ (45 g). The solutions were then incubated at RT for one week with constant stirring to facilitate complete oxidation. Samples were then diluted, repeatedly centrifuged and resuspended, and purified GO was thereby obtained.

### Temperature-sensitive PEP@GO hydrogel synthesis

A temperature-responsive polymeric hydrogel matrix was constructed by using biocompatible poly-D, l-lactic acid-polyethylene glycol-poly-d, l-lactic acid (PDLLA (1500–2000)-PEG (1000–1500)-PDLLA (1500–2000), PEP) (Shanghai Yuanye Bio-Technology Co., Ltd., China). This PEP copolymer was added to deionized (DI) water at a weight ratio of 1:5, and was incubated at 15–20 ℃ for 3 h. Then, it was stirred with a glass rod to ensure it was covered with DI water, after which samples were centrifuged at 2000 rpm at 4 ℃ until no visible debris was present. Samples were then stored overnight at 4 ℃, with the phase transition temperature being tested in the following steps.

The PEP was dissolved in a saline solution (25 wt.%) at 4 °C to make the temperature-sensitive PEP@GO hydrogel (referred to as PEP@GO). When the synthesis was completed, GO was added to yield a 3 mg/mL aqueous solution, as this concentration was sufficient to prevent GO precipitation at the final 0.75 wt.%, which could guarantee this hydrogel has modest cytotoxic effects in vitro (up to 100 g/mL) and does not cause inflammation or granuloma development in vivo. [53]. Samples were then rinsed and diluted with DI water, yielding a PEP@GO that was freeze-dried prior to subsequent characterization.

### PEP@GO characterization

An infrared thermal imaging system (MobIR Air, Guide Sensmart, Wuhan, China) was used to detect PEP and PEP@GO during the cooling—heating process (25–35 ℃). To examine hydrogels on substrate for the phase transition, PEP (25 wt. %) and PEP@GO (25 wt.%) aqueous mixes were placed as a round shaped mask (diameter = 10 mm) onto a heated silicon plate at 30 °C. Following a normal thermally responsive sol–gel transition, both hydrogels developed in situ on the heated silicon plate after roughly 20 s. To further reveal its formation and stability in situ on human skin at RT under the influence of gravity, materials were put on the human’s handback and turned vertical for 10 min at RT.

Hydrogel surface morphology and topography were examined with a scanning electron microscope (SEM, Zeiss Merlin Compact, X-Max 20 mm^2^). An Anton Paar MCR302 rheometer with a 20-mm parallel-plate arrangement was used to measure viscosity and storage/loss modulus. Hydrogel chemical structures were assessed via Fourier transform infrared spectroscopy (FT-IR, Thermo Scientific Nicolet 6700). Hundred μL of hydrogel was put on the film to observe the uniformity. The X-ray diffraction (XRD) was measured by the Rigaku SmartLab SE X-ray diffractometer (Japan). The samples were investigated in terms of contact angles (CAs) of droplets of deionized water, measured using the OCA25 instrument (Dataphysics, Germany). The influence of hydration-dehydration processes on the mechanical and morphological characteristics of the materials was tested. After gelation, freshly PEP@GO samples were immersed in water for at least three days at RT. The partly dehydrated samples were obtained by removing the swollen hydrogel disks on a Teflon sheet and placing them in a cupboard at RT for two days. The partly dehydrated samples were then rehydrated for one day in RT water. One cycle took at least three days to complete.

To explore the release kinetics of the hydrogel in vitro, 3 mL of PBS was pipetted into each 4 mL centrifuge tube with 200 μL of hydrogel at a shaking speed of 100 rpm at 37 ℃. After a predetermined time, 1 mL of the release buffer was removed and weighted after lyophilization.

### Evaluation of PEP@GO in vitro antimicrobial activity and biocompatibility

The biocompatibility of the hydrogel was determined using the Cell Counting Kit-8 (CCK-8) (Biosharp Ltd., Hefei, China). 5 × 10^3^ HUVECs (purchased from the National Collection of Authenticated Cell Cultures, Shanghai, China) were cultured in DMEM (Beijing Solarbio science & technology company, China) containing 10% sEVs-depleted fetal bovine serum (FBS) at 37 ℃ with 5% CO_2_ and cultured in different media preparations with 100 μL of hydrogel (Control: untreated, n = 6) for 0, 24, 48, and 72 h after serum-free media was added with 10 μL of CCK-8 reagent for 0.5–4 h, and the absorbance at 450 nm represents the biocompatibility.

The Live/Dead cell viability assay was also performed for hydrogel biocompatibility following the instructions (Dojindo Molecular Technologies, Japan) after seeding on the first day. The percentage of cells with positive calcein staining represents the cell viability, and the Nikon A1R confocal laser scanning microscope (Nikon, Tokyo, Japan) was used to obtain representative images.

### Diabetic wound healing experiment

The study was approved by the Institutional Animal Care and Use Committee of Union Hospital, Tongji College, Huazhong University of Science and Technology. C57BL/6 male mice were obtained from the Center of Experimental Animals, Tongji Medical College, Huazhong University of Science and Technology. A half of them were fed with streptozotocin (STZ, 60 mg/kg, Sigma) and high-fat, refined-sugar diet until being diabetic 8-week old models. Non-fasting glucose levels in diabetic mice were greater than 16.7 mmol/L in each model. Following intraperitoneal pentobarbital sodium injection (1%, 0.3 mg/kg), dorsal hair was shaved and round full-thickness cutaneous wound (diameter = 5 mm) were generated on the back of each animal proximal to the hip. Wound sites were then dressed with coverings of different groups dependent on two different mice (Diabetic Wound, DW and Normal) and two hydrogels (PEP & PEP@GO). To prevent mice from scratching the wound, medical gauze was used to cover it. The size of the infected wound was assessed after 0, 3, 7, 10, and 14 days with vernier calipers, with wound closure being measured as follows: wound closure (%) = [W_0_ − Wn]/W_0_ × 100%, where W_0_ and Wn refer to wound areas on day 0 and day n, respectively (n = 6). On day 14, wound healing outcomes were also assessed via hematoxylin and eosin (H&E) and Masson’s staining (n = 6).

### Bioinformatics analyses

To understand the potential pathomechanism for the antibacterial activity of prepared PEP@GO, we searched the GeneCards database (genecards.org/) for related genes and miRNAs using the terms “graphene oxide” and “diabetic wound”, and took the intersection [54]. To further analyze DFU alterations at the genetic level and locate the important gene or pathway, GSE80178 raw data which were significantly linked in our study’s grouping mode, were obtained from the Gene Expression Omnibus (GEO) database (ncbi.nlm.nih.gov/geo/) [55]. These data were gathered using the GPL16686 (Affymetrix Human Gene 2.0 ST Array) and processed using R Project (version 4.0.3) to identify differentially expressed genes (DEGs) between three diabetic foot skin (DFS), six DFU, and three normal foot skin samples. To normalize raw data, the R “limma” package was used, and adjusted P values (adj.p) were generated by default using the Benjamini and Hochberg false discovery rate approach. Data from two groups with the highest differentiation were logarithmically transformed, and DEGs were identified (|log Fold Change|> 2 and P 0.01). The “clusterProfiler”, “org.Hs.eg.db”, “msigdf”, and “GSEABase” R packages were used to identify enriched Gene Ontology (GO) terms with an FDR < 0.05 significance threshold. In addition, KEGG pathway and Gene Set Enrichment Analysis (GSEA) approaches were conducted and the “pheatmap” and “ggplot2” R packages were used to generate heatmaps, volcano plots, and other data visualizations.

### Regulation of miR-21 by PEP@GO

To verify the above deduction about miR-21, we double checked whether our PEP@GO could enhance miR-21. Sprague–Dawley rats (80–100 g) were used to get adipose-derived stem cells (ADSCs). Rat inguinal adipose tissue was digested and continuously shaken after it was totally minced. Finally, ADSCs were acquired. PEP@GO and PEP were cultured respectively with ADSCs in DMEM medium (Gibco, USA) supplemented with 100 U/mL penicillin, 100 U/mL streptomycin, and 10% FBS in a humidified incubator at 37 ℃ with 5% CO_2_. The mixture was centrifuged at 150,000*g* for 16 h at 4 °C in a 70ti tube (Beckman Coulter Optima TM L-100 XP). Finally, the supernatant was aspirated by pipetting, and two different groups of sEVs (PEP-sEVs and PEP@GO-sEVs) were successfully isolated and contained in a pellet at the tube bottom. SEVs morphology was assessed via TEM (EFI, TECNAI G2), DLS analyses were conducted with a Zeta VIEW instrument (Software Zeta View 8.04.02 SP2, Camera 0.703 µm/px, Particle Metrix, Germany), and sEVs surface marker expression was assessed via Western blotting. The sEVs from individuals were separately analyzed. HUVEC uptake tests were used in further in vitro and in vivo research. The qPCR was used to analyze the expression of miR-21-5p in collected sEVs samples.

Next, qPCR (quantitative polymerase chain reaction) was used to quantitatively analyze the three miRNAs in ADSCs before co-culture and in the sEVs in liquid supernatant after co-culture. Briefly, TRIpure Total RNA Extraction Reagent (ELK Biotechnology, China) was used to extract RNA from tissues, and EntiLink™ 1st Strand cDNA Synthesis Kit (ELK Biotechnology, China) was used to synthesize cDNA. The StepOneTM Real-Time PCR equipment (Life Technologies, CA, USA) was used for all the qPCR experiments. GAPDH and U6 were used to normalize mRNA and miRNA expression, respectively. To measure relative gene expression, the ΔΔCT approach was applied. Additional file 1: Table S4 contains a list of all primer sequences.

Following that, HUVEC uptake assays were carried out in a transwell polycarbonate membrane cell culture insert (Merck KGaA, Darmstadt, Germany). ADSC were co-cultivated with two types of hydrogels in the upper chamber, while HUVECs were cultured in the lower chamber, and sEVs absorption in the latter was discovered. The red PKH26 dye was utilized to label membranes and track pure sEVs (Sigma-Aldrich, USA). The sEVs were collected by ultracentrifugation and washed with 20 mL of PBS after labeling. HUVEC uptake of labeled particles was measured using immunofluorescence.

To examine the content of mi-21-5p in sEVs from ADSC triggered by hydrogels and the importance of miR-21-5p as a regulator, five HUVEC groups (n = 6) were treated with high glucose (HG), and one normal group was left untreated as a negative control. In which two HG groups were treated with wrapped relevant transfection reagents (miR-21 mimic or miR-21 NC), two HG groups were treated with PEP@GO-sEVs and PEP-sEVs, while one was untreated as the positive control. All the six groups were proceeded as following steps.

### EDU uptake assay

The biocompatibility of PEP@GO and the impact of the above experimental treatments on HUVEC cell proliferation were assessed using EDU uptake assays. HUVECs were placed in 24-well plates and treated with appropriate sEVs or miRNA formulations for 24 h. Following that, a Cell-Light EDU Apollo567 In vitro Kit (100 T) (RiboBio Co., Ltd., China) was utilized according to the instructions.

### Transwell migration assay

In a cell culture plate, appropriately treated cells were placed on the upper layer of a transwell polycarbonate membrane cell culture insert (Merck KGaA, Darmstadt, Germany). Cells that have crossed the permeable transwell membrane after 24 h were counted.

### Tube formation assay

After Matrigel coating (2 × 10^4^/well), HUVECs were introduced to 96-well plates and treated as needed for 8 h. After a 45-min incubation period, three random fields of view per sample were scanned, and both tube length and total branch point counts were quantified using ImageJ software (Java 1.8.0_112, 64-bit).

### Western blotting

Total cellular protein was extracted using lysis buffer, and protein levels were quantified using a BCA kit (ASPEN Biotechnology, Wuhan, China). Proteins were separated on a 10% SDS-PAGE before being probed with the appropriate primary antibodies for CD9, CD63 TSG101, PI3Kγ (#ab154598, Abcam, Cambridge, UK, 1:500), PIK3R1 (#4257, CST, MA, US, 1:1000), P-Akt (#4060, CST, MA, US, 1:1000), IL-17 (#sc-374218, Santa Cruz, Texas, US, 1:500), PTEN (#ab267787, Abcam, Cambridge, UK, 1:2000) and Bcl-2 (#ab182858, Abcam, Cambridge, UK, 1:1000) and then transferred to polyvinylidene fluoride (PVDF) membranes. The blots were then probed with secondary antibodies, and protein was identified using a LiDE110 scanner (Canon, Japan). GAPDH (#ab37168, Abcam, Cambridge, UK, 1:10,000) was used for normalization, and the AlphaEaseFC software was used to measure protein band density.

### Cell cycle and apoptosis assay

Cell cycle progression was determined using propidium iodide (PI) labeling, and apoptosis was determined using an Annexin V-FITC/PI apoptosis detection kit (eBioscience, USA). Flow cytometry was utilized to evaluate the cells in both techniques based on the guidelines supplied.

### Analysis of the role of the impact of sEVs on DFU healing

To examine the content of mi-21-5p in sEVs from ADSC triggered by hydrogels and the importance of miR-21-5p as a regulator, 30 diabetic male mice were randomized into five groups (six mice per group) as the diabetic wound (DW) groups, with six other normal mice as a negative control group. After being managed as a wound model, four DW groups were treated with miR-21 mimic, miR-21 NC, PEP@GO-sEVs and PEP-sEVs, while one was untreated as the positive control. On days 0, 3, 7, 10, and 14 after treatment, we looked at the wound closure ratio. On day 14, wound closure rates were evaluated, tissue integrity was assessed using H&E staining, and wound tissues were collected for qPCR analysis of miR-21-5p expression. The biocompatibility of PEP@GO and the impact of the above experimental treatments on HUVEC cell proliferation were assessed using EDU uptake assays.

### The impact of miR-21-5p on PTEN and PVT1

To assess diabetes-related interactions among miR-21-5p, PTEN, and the long non-coding RNA (lncRNA) PVT1, which could impair the self-renewal and wound healing capability of skin as previously reported [56], appropriate tools (starbase.sysu.edu.cn/, mirtarbase.mbc.nctu.edu.tw/) were used [57, 58]. Luciferase reporter assays were then used to confirm these predicted binding and regulatory reactions. In brief, reporter plasmids harboring the wild-type (WT) or mutated (Mut) versions of the 3′-UTR sequences of PTEN and PVT1 were generated, with the predicted miR-21-5p binding site being deleted in Mut constructs. Cells and a miR-21-5p mimic were then co-transfected with these constructs (10 g), and at 48 h after transfection, a Dual-Luciferase Reporter Assay Kit (Beyotime Biotechnology, Shanghai, China) was employed as directed, with data analyzed using Spark 10 M (Tecan Trading AG, Switzerland) equipment. Levels of miR-21-5p and PVT1 expression were assessed via qPCR.

To determine whether miR-21-5p could affect PTEN in HUVECs, western blotting was then used to assess gene expression using cells subjected to different treatments after targeted cells were transfected with wrapped relevant transfection reagents (NC: untreated; miR-21 mimic NC: treated by blank miR-21 mimic, blank control; miR-21 mimic: treated by miR-21 mimic).

### Assessment of the effects of PVT1 on HUVEC function

As previously stated, EDU uptake, transwell, and tube formation assays were utilized to examine the effects of PVT1 on HUVEC cell activities. The pcDNA3.1 vector was imported into the RNA mature sequence of PVT1 for lncRNA (OP-PVT1). For these experiments, cells were treated as follows: NC (negative control); PEP@GO-sEVs; PEP@GO-sEVs + OP-NC (PEP@GO-sEVs + a blank LncRNA mimic); PEP@GO-sEVs + OP-PVT1 (PEP@GO-sEVs + PVT1). Levels of miR-21-5p and PVT 1 were assessed via qPCR.

### In vivo biotoxicity testing

H&E staining of key organs, like the heart, liver, spleen, lungs, and kidneys, 14 days after hydrogel injection was used to assess the potential for nanocarrier-induced organ damage. Blood samples were also collected from control mice and mice in the PEP@GO group for routine comparisons.

### Statistical analyses

All statistical testing was done with GraphPad Prism 8.0.2 (GraphPad, CA, USA). Student's t-tests or Mann–Whitney U-tests were used to compare data. A Microsoft Excel-based online randomization program was used to allocate mice to treatment groups. Significance levels: *p < 0.05, **p < 0.01, ***p < 0.001.

## Supplementary Information

**Additional file 1:Fig S1.** The results of PEP and PEP@GO hydrogels in stability test and image uniformity; **Fig S2.** KEGG analysis of miR-21; **Fig S3.** PEP@GO hydrogel accelerated healing of diabetic wound on mice; **Table S1.** GeneCards Inferred Functionality Scores (GIFtS) and Relevance Scores of the three selected miRNAs related to the six searched keywords in Genecards database (genecards.org/); **Table S2.** miRNAs and mRNA primer sequence.

## Data Availability

The datasets generated during and/or analyzed during the current study are available from the corresponding author on reasonable request.
