# Correction: MiR-21 regulating PVT1/PTEN/IL-17 axis towards the treatment of infectious diabetic wound healing by modified GO-derived biomaterial in mouse models

**DOI:** 10.1186/s12951-022-01722-0

**Published:** 2023-01-05

**Authors:** Xi Chen, Yizhong Peng, Hang Xue, Guohui Liu, Ning Wang, Zengwu Shao

**Affiliations:** 1 Department of Orthopeadics, Union Hospital, Tongji Medical College, Huazhong University of Science and Technology, Wuhan, 430022 Hubei China; 2 National Engineering Research Center of Fiber Optic Sensing Technology and Networks, Wuhan University of Technology, Wuhan, 430070 China

**Correction: Journal of Nanobiotechnology (2022) 20:309** 10.1186/s12951-022-01516-4

Following publication of the original article [1], the authors identified an error in Figs. 2 and 5. The correct version of figures 2 and 5 are provided in this correction.

**Fig. 2:**
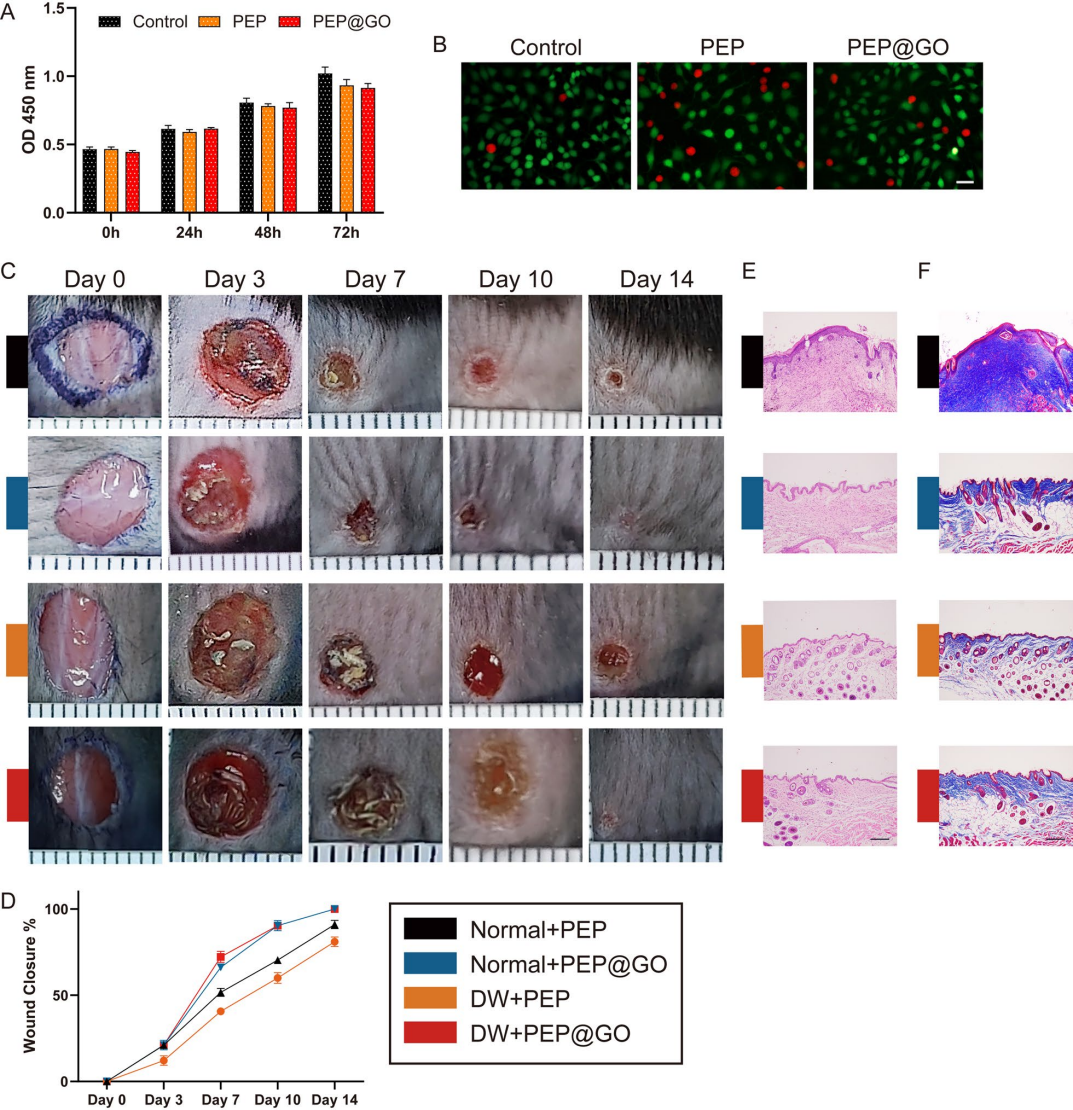
Biocompatibility of PEP@GO compared with PEP and control group. **A** CCK-8 assay results of HUVEC proliferation after treatments at 0, 24, 48 and 72 h. **B** Live/Dead staining of HUVECs cultured on day 1. Green and red fluorescent cells are viable and dead, respectively. Scale bar: 50 μm. **C**, **D** Normal and HG were constructed on the back skin of each mouse. Digital pictures were collected at Day 0, 3, 7, 10 and 14, and its statistical data, minor ticks’ interval: 1 mm. **E** H&E staining and **F** Masson staining images of skin section collected from wound area of each group at Day 14, scale bar: 200 μm. Data are the means ± SD three independent experiments. *p < 0.001

**Fig. 5:**
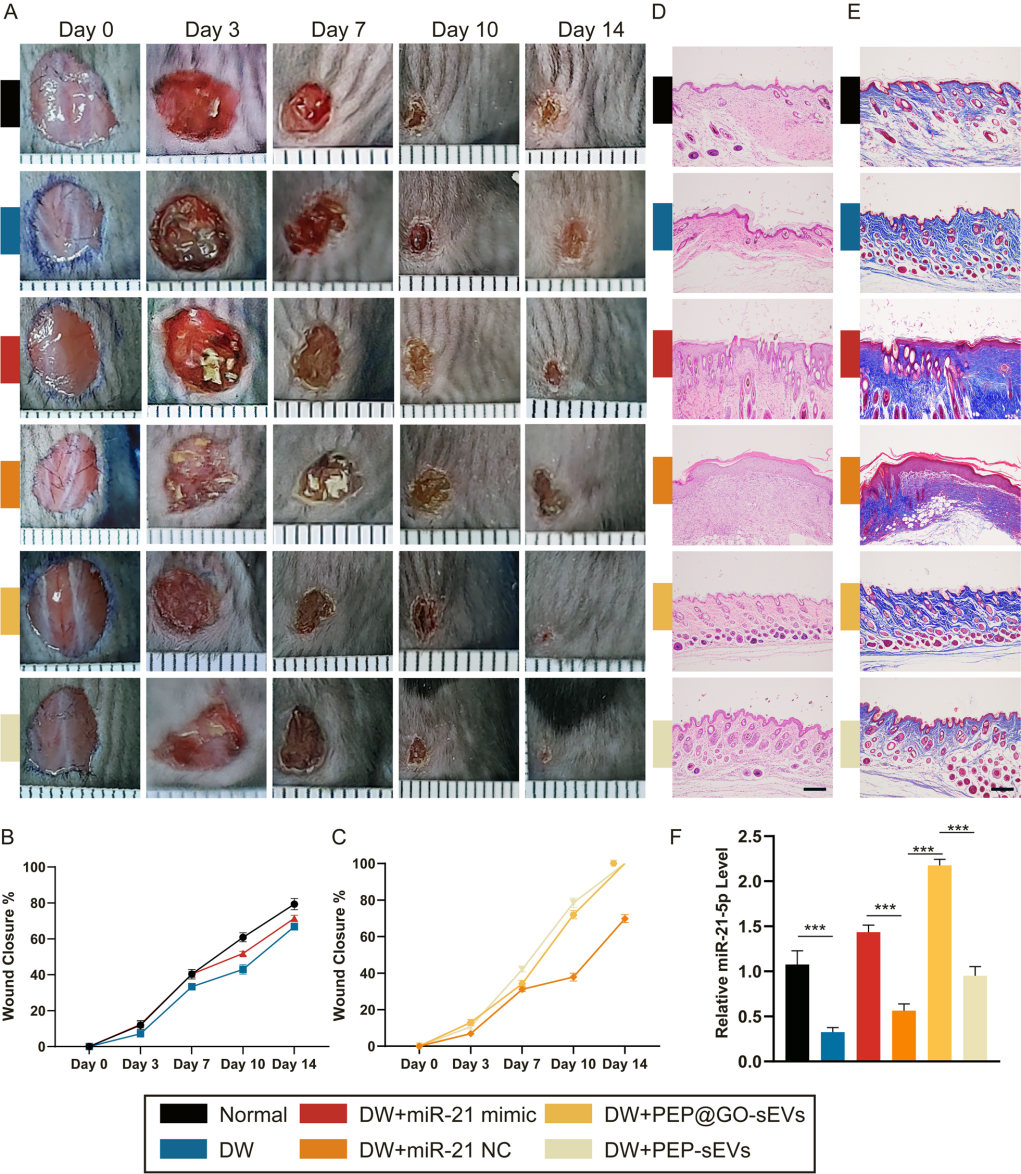
Wound healing assays were constructed on the back skin of each mouse in the six groups. **A**–**C** Representative digital pictures were collected at Day 0, 3, 7, 10 and 14 in the wound healing process and its statistical data, minor ticks’ interval: 1 mm. **D** H&E staining. **E** Masson staining images of skin sections of the six groups at Day 14. Scale bar: 200 μm. **F** qPCR results of the relative miR-21-5p level in the tissues of the six groups at Day 14. *p < 0.001

The original article [1] has been corrected.
