# Supplementary material for: MiR-21 regulating PVT1/PTEN/IL-17 axis towards the treatment of infectious diabetic wound healing by modified GO-derived biomaterial in mouse models
Source: J Nanobiotechnology. 2022 Jun 28;20:309. doi: 10.1186/s12951-022-01516-4 (PMC9238182; doi:10.1186/s12951-022-01516-4)
Supplement: Supplementary file 1 — Additional file 1:Fig S1. The results of PEP and PEP@GO hydrogels in stability test and image uniformity; Fig S2. KEGG analysis of miR-21; Fig S3. PEP@GO hydrogel accelerated healing of diabetic wound on mice; Table S1. GeneCards Inferred Functionality Scores (GIFtS) and Relevance Scores of the three selected miRNAs related to the six searched keywords in Genecards database (genecards.org/); Table S2. miRNAs and mRNA primer sequence. [file 12951_2022_1516_MOESM1_ESM.docx]

**Supporting Document**

MiR-21 regulating PVT1/PTEN/IL-17 axis towards the treatment of infectious diabetic wound healing by modified GO-derived biomaterial in mice model

Xi Chen^1^, Yizhong Peng^1^, Hang Xue^1^, Guohui Liu^1^, Ning Wang*^2^, Zengwu Shao*^1^

1. Department of Orthopeadics, Union Hospital, Tongji Medical College, Huazhong University of Science and Technology, Wuhan, Hubei, 430022, China.
2. National Engineering Laboratory for Fiber Optic Sensing Technology, Wuhan University of Technology, Wuhan 430070, China

*Corresponding author:

Email address: ningwang23@whut.edu.cn (N. Wang), [szwproo@163.com](mailto:szwpro@163.com) (Z. Shao)


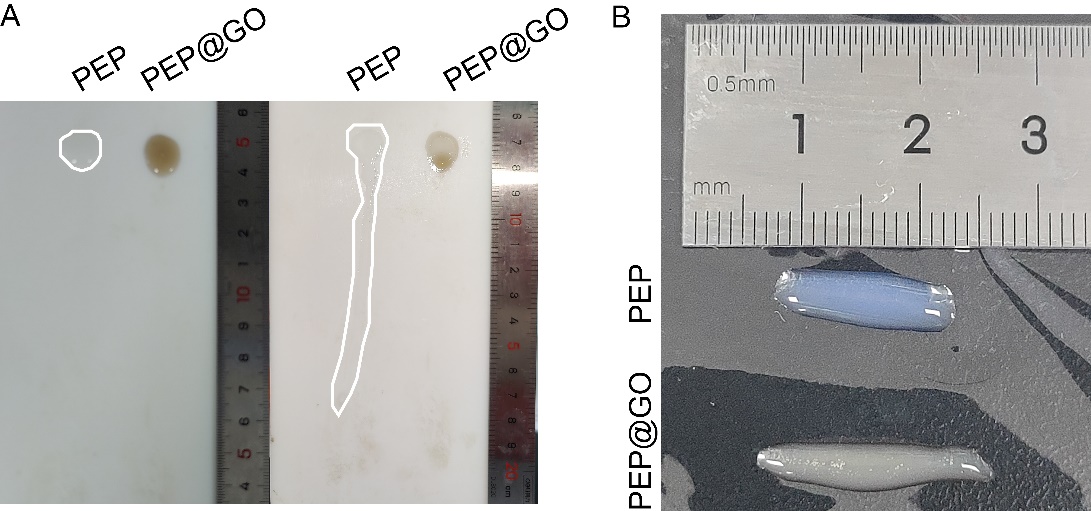


**Fig S1**. The results of PEP and PEP@GO hydrogels in stability test (A) and image uniformity (B)


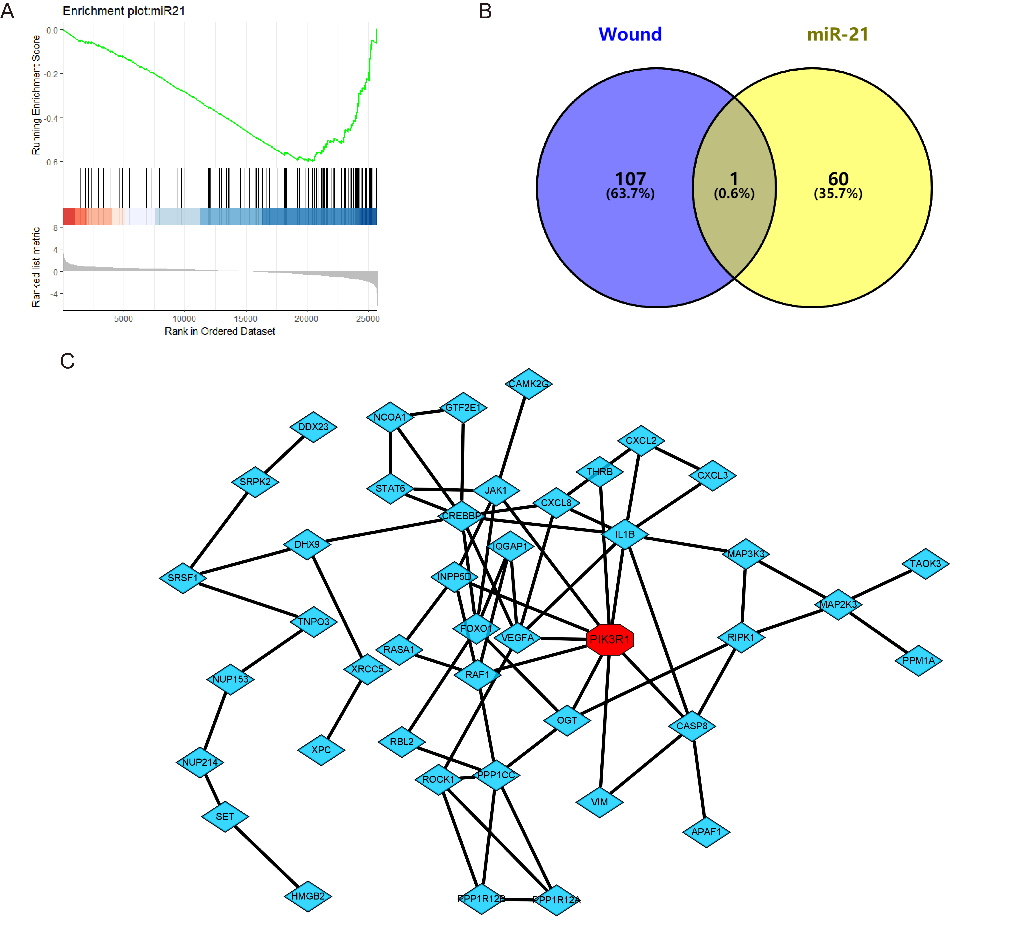


**Fig S2**. KEGG analysis of miR-21. (A) Enrichment of miR-21-5p in DFU was down-regulated compared with those in Normal. (B-C) The core target genes of miR-21-5p and wound from the obtained GSEA results were constructed as protein‑protein interaction networks using STRING's website (string-db.org, Verison 11.0) and Cytoscape 3.82, PIK3R-1 (red), the central gene that closely related with them.


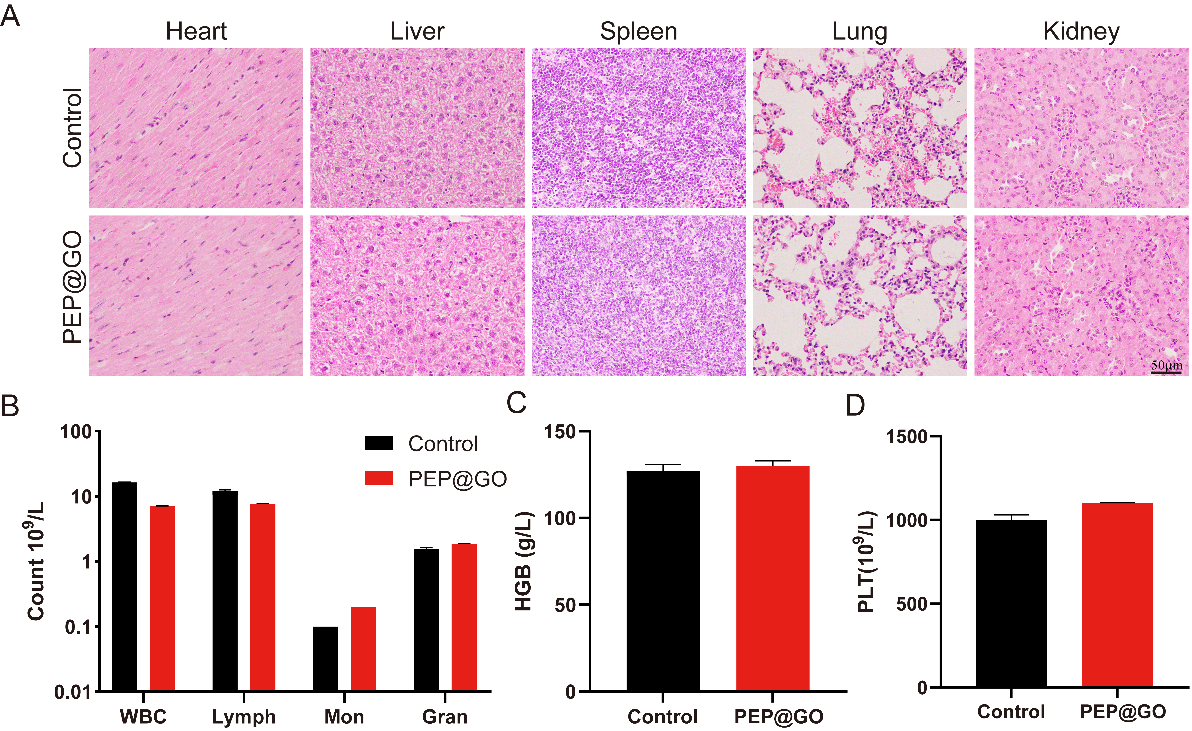


**Fig S3.** PEP@GO hydrogel accelerated healing of diabetic wound on mice. H&E stained tissue sections from major organs in mice at 14 days after the subcutaneous administration of PEP@GO, *scale bar:* 50 μm (A), Hemolysis evaluation of PEP@GO hydrogel, the major indexes of white blood count (WBC) and different kinds of cells related to immune system (B), HGB (C) and PLT (D).

| miRNA | Graphene oxide | Diabetic wounds |
| --- | --- | --- |
| miRNA-21 | 25/3.92 | 24/16.18 |
| miRNA-122 | 22/1.43 | 18/1.45 |
| miRNA-Let-7a-1 | 21/1.19 | 18/1.49 |
| miRNA-141 | 21/1.17 | 21/1.23 |
| miRNA-106a | 20/0.93 | 22/2.02 |
| miRNA-193a | 20/2.03 | 21/5.9 |

**Table S1** GeneCards Inferred Functionality Scores (GIFtS) and Relevance Scores of the three selected miRNAs related to the six searched keywords in Genecards database (genecards.org/)

| microRNAs or gene name | | Primer sequence (5’ to 3’) |
| --- | --- | --- |
| hsa –U6 | RT | AACGCTTCACGAATTTGCGT |
|  | Forward | CTCGCTTCGGCAGCACAT |
|  | Reverse | AACGCTTCACGAATTTGCGT |
| hsa-miR-21-5p | RT | CTCAACTGGTGTCGTGGAGTCGGCAATTCAGTTGAGTCAACATC |
|  | Forward | GGGGTAGCTTATCAGACTGATG |
|  | Reverse | CTCAACTGGTGTCGTGGAGTC |
| hsa-miR-122-5p | RT | CTCAACTGGTGTCGTGGAGTCGGCAATTCAGTTGAGCAAACACC |
|  | Forward | GGCCTGGAGTGTGACAATG |
|  | Reverse | CTCAACTGGTGTCGTGGAGTC |
| hsa-let-7a-5p | RT | CTCAACTGGTGTCGTGGAGTCGGCAATTCAGTTGAGAACTATAC |
|  | Forward | GGGCCTGAGGTAGTAGGTTG |
|  | Reverse | CTCAACTGGTGTCGTGGAGTC |
| mmu-miR-21 | Forward | ACGGCTTATCAGACTGATGTTGA |
|  | Reverse | CTCAACTGGTGTCGTGGAGTC |
| hsa–GAPDH | Forward | CATCATCCCTGCCTCTACTGG |
|  | Reverse | GTGGGTGTCGCTGTTGAAGTC |
| hsa–PVT1 | Forward | TCAGCACTCTGGACGGACTTG |
|  | Reverse | CTATGGCATGGGCAGGGTAG |
| hsa-PTEN | Forward | CAATATCCTTTTGAAGACCATAACC |
|  | Reverse | TGCCCCGATGTAATAAATATGC |

**Table S2**. miRNAs and mRNA primer sequence. RT: Reverse transcription.
